# Immunohistochemical Labeling of Low-Density Lipoprotein Receptor and Scavenger Receptor Class B Type 1 Are Increased in Canine Lymphoma

**DOI:** 10.3389/fvets.2018.00340

**Published:** 2019-01-11

**Authors:** Kristina Ceres, Halle Fitzgerald, Kathryn Shanelle Quiznon, Sean McDonough, Erica Behling-Kelly

**Affiliations:** ^1^Department of Population Medicine and Diagnostic Services, College of Veterinary Medicine, Cornell University, Ithaca, NY, United States; ^2^College of Agriculture and Life Sciences, Cornell University, Ithaca, NY, United States; ^3^Department of Biomedical Sciences, College of Veterinary Medicine, Cornell University, Ithaca, NY, United States

**Keywords:** sr-bi, LDL-R, cholesterol, dog, cancer, lipoprotein

## Abstract

Altered lipid metabolism is a well-documented hallmark of neoplastic transformation and impacts disease progression. Two major lipoprotein receptors, the low-density lipoprotein receptor (LDL-R) and scavenger receptor class B, type 1 (SR-BI) are overexpressed in a number of cancer types in people. These receptors serve to deliver cholesterol to the tumor cells and have been used to target drug therapies. In this study, we performed a retrospective analysis of LDL-R and SR-B1 expression in canine lymphoma using archived formalin-fixed tissue samples. Cases were immunophenotyped and classified according to World Health Organization (WHO) standards prior to immunostaining for the LDL_R and SR-B1. A total of 45 cases were evaluated; 21 high grade B (HGB), 11 low grade B (LGB), 7 high grade T (HGT), and 6 low grade T (LGT) lymphomas. One sided Wilcoxon rank sum tests were used to compare staining intensity between neoplastic and hyperplastic lymphoid tissue. The relationships between histological score and tumor grade and score and stage at presentation were assessed using non-parametric Kruskal-Wallis tests. Neoplastic lymphoid tissue expressed higher levels of both receptors compared to reactive lymph nodes. Median LDL-R score was 85.0 (interquartile range = 101.7), Median SR-B1 score was 209.0 (interquartile range 105.2). No relationship between LDL-R or SR-B1 staining score and tumor grade or phenotype was found. Serum cholesterol concentration was compared between dogs with high and low grade tumors using a two sample *T*-test, and correlations between cholesterol concentration and histological score, and between the score for the two receptors were determined using a Spearman correlation. The high expression level of these lipoprotein receptors on most of the tumors could underlie the lack of relationship between score and tumor grade. The overexpression of LDL-R and SR-B1 in canine lymphoma holds therapeutic potential particularly in dogs that overexpress one or both of these receptors, and this warrants further investigation.

## Introduction

There is a substantial and growing body of evidence documenting a role for cholesterol utilization by cells in tumor development, progression, and metastasis. Rapidly dividing cancer cells rely on high levels of uptake and/or intracellular synthesis of lipids including cholesterol to provide substrates for energy provision, to support the synthesis and structural organization of cell membranes, and as components of cell-survival signaling pathways (1–4). The functional links between cholesterol availability to tumor cells and cancer progression underlie both the recognition of a cholesterol-rich diet as a risk factor for cancer development, and the observation of low levels of serum cholesterol commonly seen in patients with bowel, lung, prostate, head and neck cancers, and hematopoietic cancers (5–7). Pharmacologic blockade of cholesterol synthesis and cholesterol influx into tumors cells are both emerging as therapeutic options in combating human cancer.

Lipoprotein receptors provide conduits for cholesterol delivery to cells. The scavenger receptor class B, type I (SR-BI) binds high density lipoproteins (HDL) and facilitates the transport of cholesteryl esters from HDL into cells (8). Apolipoprotein B in low density lipoprotein (LDL) binding to the LDL receptor (LDL-R) targets the lipoprotein-receptor complex to the endocytic pathway transporting cholesterol directly into the cell (7). The SR-B1 receptor is aberrantly or overexpressed in a number of malignancies including; prostatic carcinoma, breast cancer, renal cell carcinoma, and hematopoietic tumors (8–11). Similarly, high LDL-R expression has been documented in breast cancer cell lines (12). Alterations in the expression levels of these receptors often co-exist with changes in the lipoproteins they shuttle from the blood into the tumor cells.

Studies have documented aberrant lipoprotein concentrations in people with a variety of hematologic cancers. In a study of 25 non-Hodgkin's lymphoma patients, all patients exhibited increased very low-density lipoprotein, and decreased high density lipoproteins (HDL) (13). An additional study on 530 patients found that both low-density lipoprotein (LDL) and HDL levels were lower in patients with hematological malignancies than in patients without malignancies (14). *In vitro* experiments support LDL-induced changes in the cholesterol content of leukemic cells as well as their signaling and proliferative responses (15). Recently, low HDL was shown to be an independent poor prognostic indicator in extranodal natural killer/T cell lymphoma (16). Despite the growing body of evidence that the dog is a viable model for human lymphoma, the links between lipid metabolism, cholesterol, and cancer development and progression in the dog are poorly characterized at this time (17). The need for this knowledge is heighted by the fact that non-Hodgkin lymphoma makes up 83% of all canine hematopoietic cancer, with diffuse large B-cell lymphoma (DLBCL) being the most common subtype (18, 19).

In this study, we hypothesized that the SR-BI and LDL-R would be expressed on neoplastic lymphocytes and that expression levels would correlate to tumor grade. To test our hypothesis, we performed a retrospective study using archived formalin-fixed tissue samples and immunohistochemical staining. Cases were immunophenotyped and classified according to World Health Organization (WHO) standards. Immunohistochemical staining intensity was scored and compared across tumor grade. Reactive lymph nodes were included in the analysis for comparison.

## Materials and Methods

### Case Selection

Medical records of animals admitted to the Cornell University Hospital for Animals were searched from 2001 to 2015 for confirmed cases of multicentric lymphoma. Cases were included if archived material was anticipated to be sufficient to complete the staining procedure with all appropriate controls and excluded if the dogs had been treated with chemotherapeutic agents including corticosteroids prior to collection of the tissue. The exclusion of dogs that had been given corticosteroids is critical in any study evaluating lipoprotein receptors, as these drugs modulate intracellular lipid metabolism which in turn affects expression of these receptors (20–22). Tumor grading according to the World Health Organization classification standards was performed on all cases as part of the diagnostic work-up. Grading of all cases was independently confirmed by a single pathologist (SM) who was aware only of immunophenotype. Cases were placed into one of four categories for the purposes of this study: low-grade B-Cell (LGB), high-grade B-cell (HGB), low-grade T- Cell (LGT), and high-grade T-Cell (HGT). Six reactive lymph node cases were also selected as controls. Total serum cholesterol concentrations were recorded if a chemistry panel was run within 3 days of the biopsy collection date.

### Tissue Collection

Client consent was obtained as part of the diagnostic evaluation of the patients. The tissue biopsies were collected and processed as per standard procedures at the Cornell University Hospital for Animals. Biopsies are routinely fixed for a standard 24 h in 4% paraformaldehyde, processed, and embedded in paraffin as per the standard protocol at the Histology Laboratory of Cornell University College of Veterinary Medicine, Animal Health Diagnostic Center. Hematoxylin-eosin stained sections were reviewed for tumor grading (SM). Six additional 4 μm sections were cut and placed on charged slides for immunohistochemical processing. Liver tissue was collected from the Cornell University tissue bank and processed in the same manner.

### Samples for Method Validation

Canine liver tissue, expected to express both receptors, was used for initial method optimization and antibody titration (23, 24). The livers were collected from research beagles as part of Cornell's tissue sharing program. Antibody tissue-specificity was confirmed using a canine tissue array including spleen, kidney, heart, pancreas, adrenal gland, esophagus, stomach, duodenum, jejunum, ileum, colon, thyroid gland, pituitary gland, testis, prostate, lung, aorta, salivary gland, mesenteric lymph node, tonsil, frontal lobe, and skin. An isotype-matched antibody was used as a negative control.

### Immunohistochemical Labeling of LDL-R and SR-BI

The sections were deparaffinized by heating for 45 min at 65°C followed by three successive baths of D-limonene at 3 min each. Sections were then dried at room temperature until they turned white. Then they were placed in 10 mM Tris, 1 mM EDTA solution pH 9.0 for antigen retrieval (Invitrogen, Carlsbad CA cat# 50-187-83) and microwaved in a pressure cooker for 5 min followed by placing sections in phosphate buffer saline pH 7.4 (10 mM PO${}_{4}^{3-}$, 137 mM NaCl, and 2.7 mM KCl) (PBS) for 10 min at room temperature. Endogenous peroxidase activity was quenched by incubating sections for 5 min with 3% H_2_O_2_ in methanol at room temperature. Sections were washed 3X in PBS for 10 min at room temperature. Next non-specific binding was blocked by Ready-To-Use horse serum (Vector laboratories, Burlingame CA Cat S-2000) for 30 min at room temperature. This was followed by primary antibody incubation anti-LDL-R (Abcam 30532 Rabbit polyclonal: 1:50, 0.25 mg/ml working concentration) and anti-SR-BI (ThermoFischer Scientific PA1-16788 Rabbit polyclonal: 1:300, 0.0034 mg/ml working concentration) for 60 min at 37°C. Next sections were incubated with an anti-rabbit polymer conjugated to horseradish peroxidase (Vector laboratories, Burlingame CA) for 30 min at room temperature. Sections were then placed in PBS for 10 min and then developed with DAB chromogen solution (Vector laboratories, Burlingame CA). Sections were counterstained with hematoxylin, air-dried and cover slipped. Controls included liver and lymph node sections incubated with an isotype control (ThermoFischer Scientific 31235: 1:50, 0.23 mg/ml working concentration) and no primary antibody. Tissue arrays were processed using the same protocol. After optimization, slides from cases were run in batches including 3 sections from each lymph node and liver sample. The lymph node and liver slides were incubated with anti-LDL-R, anti-SR-B1, or an isotype antibody using identical conditions.

### Evaluation of LDL-R and SR-BI Staining

Sections were evaluated using a subjective visual scale by two scorers. Five hundred cells in five 500X visual fields per section were evaluated for cell staining intensity. Cells were placed in one of three categories: no stain, weakly stained, or strongly stained. The average number of cells in each category per section was recorded. A numeric value between 0 and 300, the H-score, was assigned to each section using the following equation:

$$\begin{array}{l} {\left(Average\;percent\;strongly\;stained\right)}^{*}3\;\\ +\;{\left(average\;percent\;weakly\;stained\right)}^{*}2=scaled\;score \end{array}$$

All cases were scored by two individuals and the average of the scores was used in comparative analysis.

### Statistical Analysis

The H-score for each section was used to evaluate differences in staining characteristics. The intraclass correlation coefficient was calculated using the *irr* package in R to assess rater score agreement. H-scores were evaluated for normality using Shapiro-Wilks tests (LDLr score W = 0.93, *p* = 0.01, SR-B1 score W = 0.93, *p* = 0.07). Since SR-B1 score was not normally distributed, non-parametric tests were used for all comparisons. A monotonic relationship between grade and score was not observed, so a correlation was not assessed. The neoplastic and reactive lymphoid tissue H-scores were compared using a one-sided Wilcoxon rank sum tests. Relationships between H-scores and tumor grade, phenotypes and clinical stage at presentation were evaluated using Kruskal-Wallis tests. Serum cholesterol concentrations in dogs with high grade tumors was compared to that in dogs with low grade tumors using a two sample *T*-test. Correlations between cholesterol concentration and H-score, and between the H-score for the two receptors were determined using a Spearman correlation. Significance was defined as *p* < 0.05. All statistical analysis was conducted in Rstudio version 1.1.419. R version 3.3.4.

## Results

### Antibody Validation

**Figure 1:**
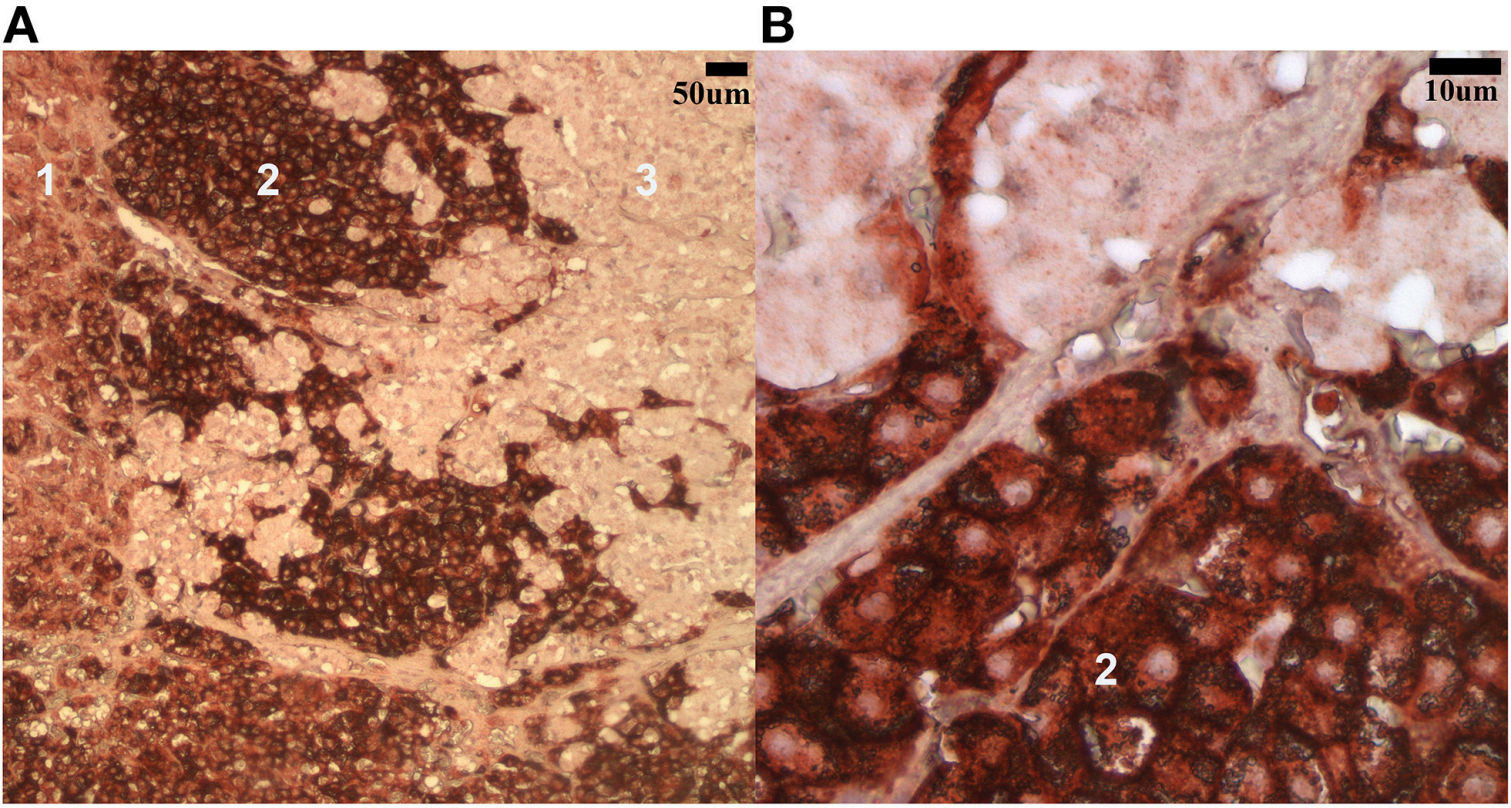
Low **(A)** and high **(B)** magnification (100x and 500x, respectively) images of the steroidogenic cells in the canine adrenal gland labeled with the SR-B1 antibody. The most intense staining is noted in the fasciculata layer (2) while less intense positive staining is seen in the zona glomerulosa (1) and zona reticularis (3).

**Figure 2:**
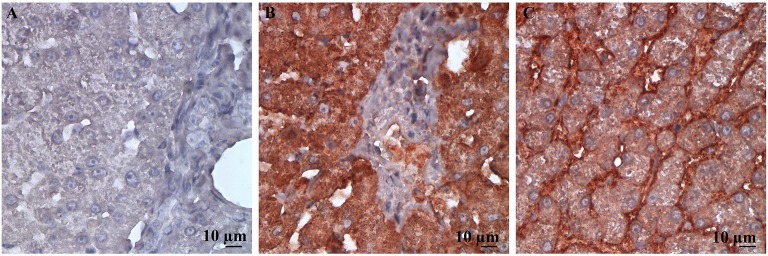
High power (500X) images of canine liver tissue labeled immunohistochemically with **(A)** the isotype control antibody; **(B)** LDL-R antibody; and **(C)** SR-B1 antibody. The staining pattern for LDL-R is diffusely strongly positive in hepatocytes imaged in **(A)**, as expected. Similarly, staining pattern is membranous and weakly diffuse in the cytoplasm of hepatocytes in **(B)**, an expected finding for SR-B1.

The staining patterns for both antibodies were as expected on the tissues of interest in the tissue array. A clear gradient of SR-B1 expression in the anatomical zones of the adrenal gland was evident, as has been reported in human adrenal glands (25) (Figure 1). The pattern of staining in hepatic tissue was as anticipated for both the LDL-R and SR-B1 antibodies (23) (Figure 2). Tissues incubated with the isotype control antibody were universally negative aside from non-specific staining of the splenic red pulp and plasma cells.

### Patient Data

A total of 45 cases met all the case selection criteria. Adequate data were available to retrospectively determine the clinical stage at presentation for 39 of the 45 cases and distribution was as follows: 11 stage 5, 10 stage 4, 17 stage 3, 1 stage 1. Clinical sub-stage was not included in the analysis due to the lack of standardized criteria (26) 1 case was removed due to poor LDL-R immunohistochemical staining on the paired liver control tissue, and 11 cases were not evaluated for SR-B1 staining due to limited tissue availability in the archived block. The patient population included 20 spayed females, 19 castrated males, 4 intact males, and 2 intact females. The median (range) of patient age was 9.3 (4–15) years. The majority of dogs were mixed breed (22) with golden retrievers being the most numerous single breed represented (6). Other breeds included: Labrador retriever (3) miniature schnauzer, greyhound, border collie, smooth fox terrier, shih tzu, springer spaniel, Siberian husky, miniature pinscher, Rottweiler, German shepherd, boxer, great dane, airedale terrier, and pug. The grades of the tumors in the cases successfully evaluated were distributed as follows: 21 HGB, 11 LGB, 7 HGT, and 6 LGT. The cases used to evaluate reactive processes in the included benign follicular hyperplasia (3), sinus histiocytosis (1), paracortical T-cell expansion (1) and follicular dysplasia characterized by a loss of mantle zones with otherwise retained architecture (1). The majority of patients were lost to follow-up (28) or euthanized (13) whereas 4 died at home. Three of the deaths at home were attributed to progression of disease and 1 the result of immune-mediated hemolytic anemia.

### LDL-R and SR-B1 Expression

**Figure 3:**
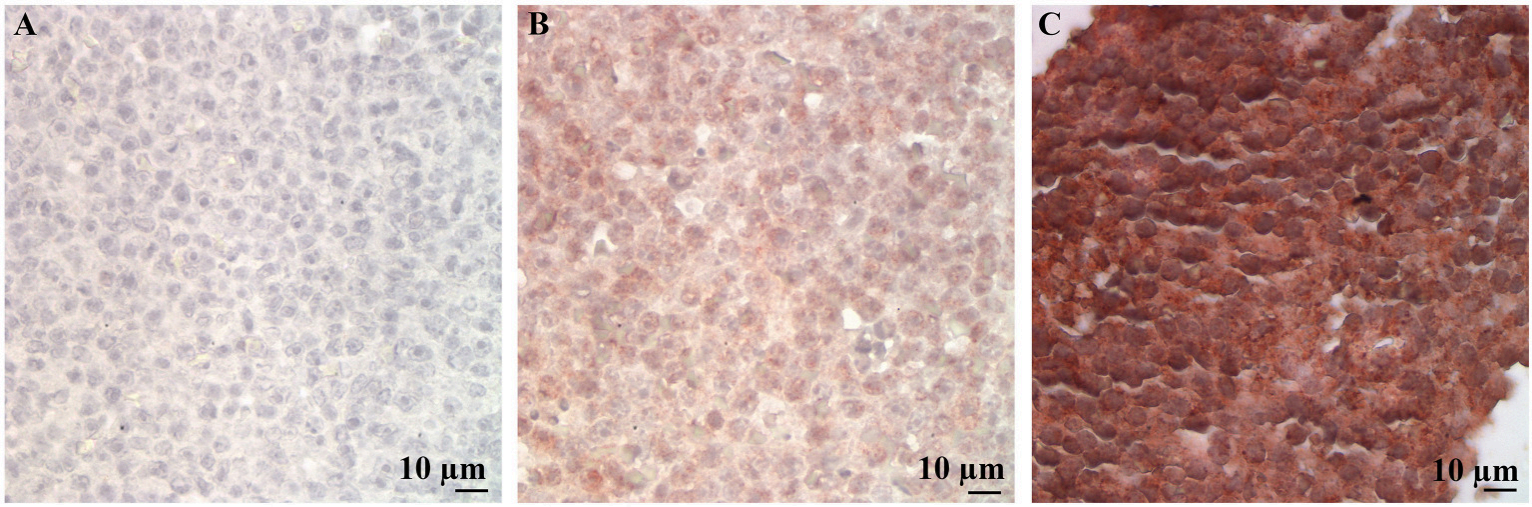
Representative LDL-R and SR-B1 staining of canine lymphoma cases and controls. High power (500X) images of a single representative case demonstrating no staining with the isotype control **(A)**, weak staining for LDL-R **(B)** and strong staining for SR-B1 **(C)**.

**Figure 4:**
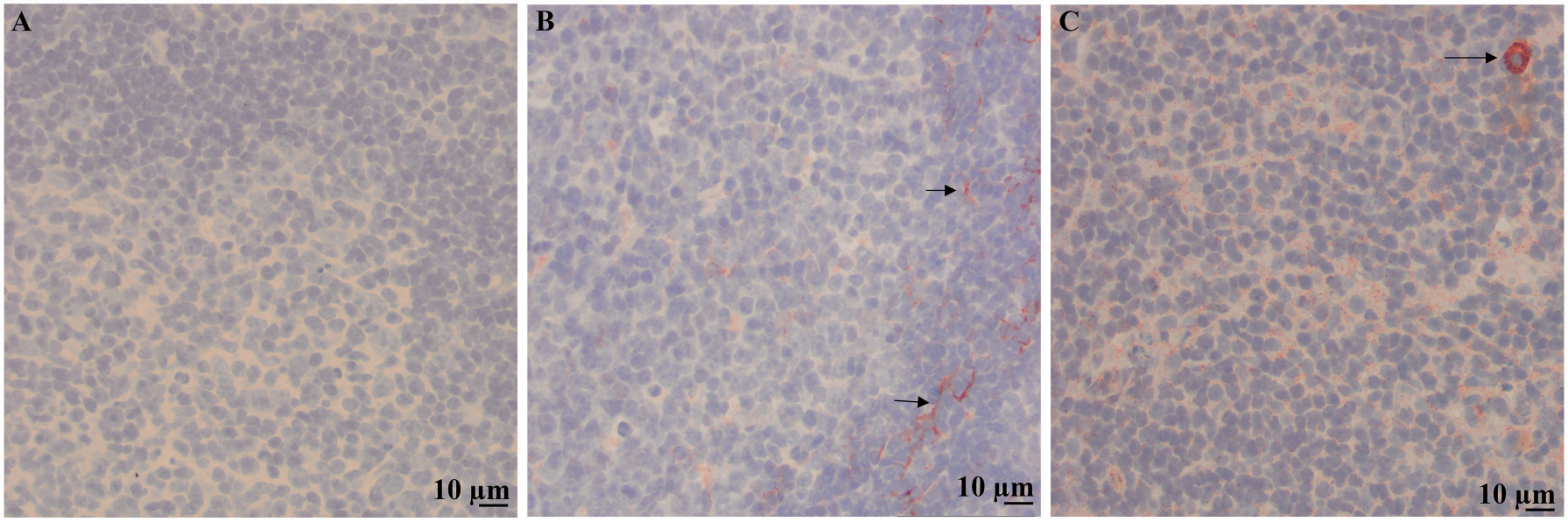
Immunolabeling of a reactive lymph node with benign follicular hyperplasia. 200X images of representative sections demonstrating no staining with the isotype control **(A)**, staining for LDL-R on cytoplasmic processes of dendritic cells in the mantle zone of a follicle (arrows) **(B)** and weak punctate staining of low numbers of lymphocytes and strong staining of macrophages (arrow) for SR-B1 **(C)**.

Immunohistochemical labeling of neoplastic lymphocytes for LDL-R and SR-B1 was universally positive but variable in intensity across the individual cases evaluated. Representative images are shown in Figure 3. The lymph nodes with reactive lymphoid hyperplasia displayed minimal positive staining with low numbers of the reactive lymphocytes immunolabeled for SR-B1. Follicular dendritic cells and macrophages were strongly immunolabeled for LDL-R and SR-B1, respectively (Figure 4).

**Figure 5:**
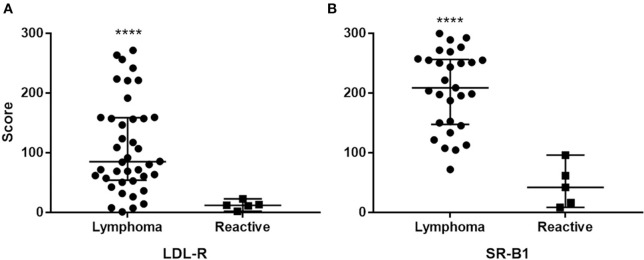
Distribution of staining intensity score among cases and controls (reactive lymph nodes) stained with LDL-R **(A)** and SR-B1 **(B)**. Solid bars represent the median scores. ****Different from reactive node score *p* < 0.0001.

The intraclass correlation coefficient among scorers was 0.59, 95%CI [0.41, 0.72], which indicates moderate agreement. For neoplastic tissue the median LDL-R score was 85.0 (interquartile range = 101.7), median SR-B1 score was 12.0 (interquartile range 44.8). For reactive lymphoid tissue the median LDL-R score was 12 (interquartile range 11.4), median SR-B1 score was 42 (interquartile range 66.1). LDL-R or SR-B1 staining score were not significantly different across tumor grade, phenotype, or clinical stage at presentation (*p* > 0.1 for all comparisons). The scores for the two receptors showed no correlation (Spearman *r* = 0.187, *p* = 0.3). Scaled scores for reactive lymph nodes and cases were found to differ for both LDL-R (*W* = 186, *p* < 0.0001) and SR-B1 (*W* = 144, *p* < 0.0001) staining score (Figure 5).

#### Serum Cholesterol Concentration

**Figure 6:**
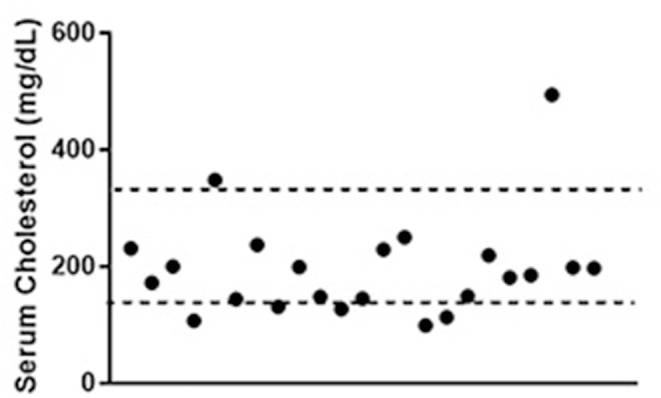
Total serum cholesterol concentrations in patients with lymphoma. Dotted lines represent the limits of the reference interval at the study institution (138–332 mg/dL). Data were available for 23 out of the 45 dogs included in the study. Values for 5 of the dogs (21%) were below the reference interval.

Total serum cholesterol concentration data were available for 23 of the patients (mean of 195.8 mg/dL, range 99–494 mg/dL) and was below the reference interval established at Cornell University's Clinical Pathology laboratory (138–332 mg/dL) in 5/23 (mean 115.4 mg/dl, range 99–131 mg/dL). No clinically identified gastrointestinal disease was present in these 5 dogs. The cholesterol concentration tracked near the lower half of the interval in the majority of the dogs (Figure 6). The median cholesterol concentration was no different in dogs with high grade tumors compared to those with low grade tumors (*p* = 0.15). No correlation was found between tumor grade, phenotype or clinical stage at presentation and serum cholesterol concentration.

## Discussion

This is the first study investigating LDL-R and SR-BI immunolabeling in canine cancer. We demonstrated that canine lymphoma cells express both these receptors at higher levels than non-neoplastic lymphocytes within reactive lymphoid tissue. We did not detect a correlation between expression level semi-quantified by immunohistochemistry of either receptor and tumor grade. These findings raise the possibility that LDL-R and SR-B1 may provide novel targets for chemotherapeutic drugs in the dog.

The dependence tumor cells have on SR-B1-mediated cholesterol delivery has been demonstrated in a number of studies. Disruption of this pathway by inhibiting the expression of SR-B1 reduces the proliferation and invasive behavior of human renal cell carcinoma and nasal pharyngeal carcinoma cell lines (27, 28). Synthetic HDL nanoparticles (HDL-NP) interfere directly with cholesterol delivery to cells and are highly efficacious in targeting human hematopoietic tumors lines (29–31). HDL nanoparticles have also proven effective in depleting cellular cholesterol levels and blocking proliferation pathways in medulloblastoma and Ewing sarcoma cell lines, two notoriously difficult to treat pediatric cancers (32). Similar HDL-NP mediated inhibition of growth, motility, and metastasis of nasopharyngeal cancer cells have been documented (28) An HDL-NP approach could be explored as a way to target SR-BI expressing neoplastic lymphocytes in canine lymphoma patients.

Nanoparticles synthesized to resemble LDL and target LDL-R have also been evaluated and found to have high toxicity to melanoma cells and reduced off-target effects (33). Lipid-encapsulation of drugs directs their delivery to LDL-R and other lipid-uptake receptors. Previous studies comparing lipid-encapsulated doxorubicin to the standard drug in canine lymphoma cases did not detect an appreciable difference (34). However, sample size was small and no selection criteria relative to lipid metabolism or expression of lipid uptake receptors was included. Our results indicate that expression of the LDL-R in canine lymphoma is variable. Thus, the ability to detect an improved efficacy with a lipid encapsulated drug could be masked if patients with higher levels of the receptor are not prospectively selected. Selecting dogs that have tumors with the highest level of expression for this type of targeted treatment may yield different results. A flow cytometric assay to quantify expression of these receptors is currently being developed in the Cornell Clinical Pathology laboratory, and could provide a means to screen patients and identify those most likely to benefit from drugs targeting these receptors. Additionally, the overall higher intensity of SR-B1 expression suggests that this receptor may prove to be a better therapeutic target than the LDL-R.

The lack of correlation between LDL-R or SR-BI expression and lymphoma tumor grade could be the result of non-static expression of these cellular receptors in tumors. In human breast cancer patients, expression of genes regulating estrogen signaling in breast tumorigenesis was dependent on circadian rhythms (35). The regulation of LDL-R and SR-B1 in the dog is poorly characterized. However, in rodents both diet and hormone treatment impact expression of these receptors. In mice, cholesterol feeding increases brain SR-BI mRNA levels but has no impact on hepatic expression levels (36). In rats, the mRNA levels for both these receptors is not impacted by diet nor does it display a diurnal variation (37). The effects of hormones on the lipoprotein receptors is also divergent across species. Estrogen treatment of mice leads to a decrease in hepatic SR-BI, but an increase brain SR-BI mRNA. Contrast this to rats which show an estradiol-induced decrease SR-BI in liver, but increase in the adrenal glands (36). Since we only evaluated one biopsy per case, we do not know if the expression of these receptors is subject to hormonal or dietary influences in the dog. These factors should be evaluated as lipid metabolism is further investigated in the context of canine cancer.

The widespread use of inhibitors of cholesterol biosynthesis to treat cardiovascular disease led to the recognition of an epidemiological link between their use and decreased cancer risk or cancer recurrence in people (38–42). *In vitro* studies exploring cholesterol blockade have been promising. For example, treatment of chemoresistant leukemia blasts with statins, which inhibit the rate limiting step in cholesterol synthesis, restores chemosensitivity (43). This approach has been therapeutically exploited in treatment of a number of clinical trials (44, 45). These trials have generated mixed results further highlighting the need for reliable biomarkers for the patients most likely to benefit from a cholesterol-targeted approach (46).

Sample size in the current study was limited by both stringent inclusion criteria and a temporal shift toward flow cytometric immunophenotyping in preference to excisional biopsy and evaluation of lymph nodes in patient care at the study institution. High grade B-cell tumors account for approximately 50% of canine lymphomas and T-cell lymphomas are much less prevalent (17). Thus our evaluation of T cell lymphomas was not surprisingly very limited in this study.

Lipid metabolism and its impact on the development and progression of hematopoietic tumors in the dog is understudied. Our findings demonstrate that neoplastic lymphocytes increase the expression of receptors known to deliver cholesterol to cells. The biological impact of this findings and the potential to therapeutically exploit it are areas worthy of furtherer investigation.

## Author Contributions

KC performed IHC staining, performed cell counts, analyzed data, drafted manuscript. HF and KQ performed cell counts and helped analyze data. SM provided histological grade. EB-K was responsible for study design, project oversight, and final manuscript preparation.

### Conflict of Interest Statement

The authors declare that the research was conducted in the absence of any commercial or financial relationships that could be construed as a potential conflict of interest.
